# Domestic Pigs Have Low Susceptibility to H5N1 Highly Pathogenic Avian Influenza Viruses

**DOI:** 10.1371/journal.ppat.1000102

**Published:** 2008-07-11

**Authors:** Aleksandr S. Lipatov, Yong Kuk Kwon, Luciana V. Sarmento, Kelly M. Lager, Erica Spackman, David L. Suarez, David E. Swayne

**Affiliations:** 1 Southeast Poultry Research Laboratory, Agricultural Research Service, United States Department of Agriculture, Athens, Georgia, United States of America; 2 National Animal Diseases Center, Agricultural Research Service, United States Department of Agriculture, Ames, Iowa, United States of America; St. Jude Children's Research Hospital, United States of America

## Abstract

Genetic reassortment of H5N1 highly pathogenic avian influenza viruses (HPAI) with currently circulating human influenza A strains is one possibility that could lead to efficient human-to-human transmissibility. Domestic pigs which are susceptible to infection with both human and avian influenza A viruses are one of the natural hosts where such reassortment events could occur. Virological, histological and serological features of H5N1 virus infection in pigs were characterized in this study. Two- to three-week-old domestic piglets were intranasally inoculated with 10^6^ EID_50_ of A/Vietnam/1203/04 (VN/04), A/chicken/Indonesia/7/03 (Ck/Indo/03), A/Whooper swan/Mongolia/244/05 (WS/Mong/05), and A/Muscovy duck/Vietnam/ 209/05 (MDk/VN/05) viruses. Swine H3N2 and H1N1 viruses were studied as a positive control for swine influenza virus infection. The pathogenicity of the H5N1 HPAI viruses was also characterized in mouse and ferret animal models. Intranasal inoculation of pigs with H5N1 viruses or consumption of infected chicken meat did not result in severe disease. Mild weight loss was seen in pigs inoculated with WS/Mong/05, Ck/Indo/03 H5N1 and H1N1 swine influenza viruses. WS/Mong/05, Ck/Indo/03 and VN/04 viruses were detected in nasal swabs of inoculated pigs mainly on days 1 and 3. Titers of H5N1 viruses in nasal swabs were remarkably lower compared with those of swine influenza viruses. Replication of all four H5N1 viruses in pigs was restricted to the respiratory tract, mainly to the lungs. Titers of H5N1 viruses in the lungs were lower than those of swine viruses. WS/Mong/05 virus was isolated from trachea and tonsils, and MDk/VN/05 virus was isolated from nasal turbinate of infected pigs. Histological examination revealed mild to moderate bronchiolitis and multifocal alveolitis in the lungs of pigs infected with H5N1 viruses, while infection with swine influenza viruses resulted in severe tracheobronchitis and bronchointerstitial pneumonia. Pigs had low susceptibility to infection with H5N1 HPAI viruses. Inoculation of pigs with H5N1 viruses resulted in asymptomatic to mild symptomatic infection restricted to the respiratory tract and tonsils in contrast to mouse and ferrets animal models, where some of the viruses studied were highly pathogenic and replicated systemically.

## Author Summary

Highly pathogenic avian influenza A viruses of H5N1 subtype have spread through Eurasia and Africa with continuing cases of human infection, suggesting the potential to become a pandemic influenza virus. Pigs which are susceptible to infection with both human and avian influenza A viruses are one of the natural hosts where a pandemic virus could be produced. In this study, we characterized in a pig model the infection caused by four H5N1 virus strains isolated from humans, poultry and wild birds. We demonstrated that exposure of pigs through the nose with H5N1 viruses or consumption of meat from infected chickens resulted in infection with mild weight loss. In contrast to mouse and ferret animal models where some of viruses were highly pathogenic and replicated in multiple organs, replication of H5N1 viruses in pigs was restricted to the respiratory tract, mainly to the lungs, and tonsils. Mild to moderate bronchiolitis and pneumonia were observed in the lungs of infected animals. Our results demonstrated that domestic pigs had low susceptibility to infection and disease with highly pathogenic H5N1 influenza A viruses.

## Introduction

The genus *Influenzavirus A* (i.e. influenza A virus) contains individual virus strains which have infected a broad spectrum of avian and mammalian species. While wild aquatic birds are the primordial reservoirs for all influenza A virus genes and subtypes, distinct genetic lineages have become established in humans, horses, and pigs [1],[2]. Viruses of 3 different subtypes, H1N1, H3N2, and H1N2, are circulating in swine worldwide (reviewed in [3],[4]). The origin and nature of swine influenza viruses vary on different continents. Most swine influenza A viruses are reassortants containing various combinations of genes originating from human, avian and swine influenza A viruses [3],[4]. This emphasizes that pigs are susceptible to both human and avian influenza viruses. Such susceptibility could possibly be explained by the presence of cell surface receptors for both human and avian influenza viruses on the epithelium of pig upper respiratory tract [5]. These features enable pigs to be a possible intermediate host or “mixing vessel”, for the generation of pandemic influenza viruses through reassortment [6],[7]. The 1957 and 1968 pandemic influenza viruses were reassortants which contained human and avian influenza virus genes [8],[9]. However, there is no proof for a role of pigs in the generation of these pandemic viruses. The 1918 H1N1 “Spanish” pandemic influenza virus appears to have entered both human and pig populations, although the epidemiological evidence favors humans as the initial host [10]. There are a number of reports of human infection with influenza viruses of swine origin (reviewed in [4]). Thus, it is obvious that pigs are an important link in the ecology of influenza A viruses and could be a possible source of origin for human pandemic influenza.

Highly pathogenic avian influenza (HPAI) viruses of the H5N1 subtype are zoonotic agents that present a continuing threat to both veterinary and public health (reviewed in [11]). Between 1996 and 2003, H5N1 HPAI viruses were isolated from poultry in Southern China [12],[13] and Vietnam [14], and occasionally caused severe disease in humans [13],[15],[16]. The situation changed in late 2003–2004, when the H5N1 viruses expanded their geographic range, resulting in unprecedented epizootics in poultry and new human cases in eastern and southeastern Asia [17],[18]. In May 2005, an H5N1 disease outbreak in migratory waterfowl occurred at Qinghai Lake in Western China, and signaled a possible wild bird component to the spread of H5N1 in the region [19],[20]. During 2005–2007, H5N1 viruses spread throughout Asia, Europe, Middle East, North and West Africa [21]. Outbreaks in poultry and cases of human H5N1 disease with a high case fatality rate have continued through 2007 and into the beginning of 2008 [21],[22].

The endemicity of H5N1 HPAI virus in village poultry of Eurasia and Africa [23], and the continuing appearance of individual human cases have created a situation that may facilitate pandemic emergence. However, to date, most cases of human infection with H5N1 HPAI viruses have occurred through close contacts with infected village poultry [24]. Human-to-human transmission of H5N1 viruses has been inefficient and limited [11],[24],[25],[26]. The transmissibility of H5N1 viruses in mammalian models, such as pigs and ferrets, has been inefficient [27],[28],[29]. There are potentially two ways for H5N1 HPAI viruses to acquire efficient interhuman transmissibility: 1) genetic reassortment with circulating human influenza A viruses or 2) the accumulation of mutations during adaptation in mammalian hosts [30],[31],[32]. Potentially, pigs could be the natural host where either of these events could occur.

There are a number of reports of natural H5N1 HPAI virus infection of animals taxonomically belonging to the order *Carnivora* (i.e. domestic cats, tigers, leopards, dogs and stone martens) [33],[34],[35],[36],[37]. Data on isolation of H5N1 viruses from pigs (*Sus scrofa*, family *Suidae*, order *Artiodactyla*) has been very limited [38],[39],[40]. Sero-epidemiological studies of Choi and co-authors [27] show that 0.25% (8 of 3,175) of pig sera collected at slaughterhouses in Vietnam in 2004 were seropositive from H5N1 virus infections. Studies of serum samples collected from pigs during H5N1 poultry outbreaks in Korea during the winter season of 2003 did not reveal any evidence of H5N1 HPAI virus infection [41]. No virological or serological confirmation of infection was observed in miniature pigs after experimental infection with A/chicken/Yamaguchi/7/04 and A/duck/Yokohama/aq-10/03 (H5N1) viruses [42]. Inoculation of Yorkshire white piglets with two Hong Kong 1997 H5N1 HPAI isolates, and two Vietnamese and two Thai 2004 isolates resulted in mild to moderate infection restricted mainly to the respiratory tract [43],[27].

**Figure 1:**
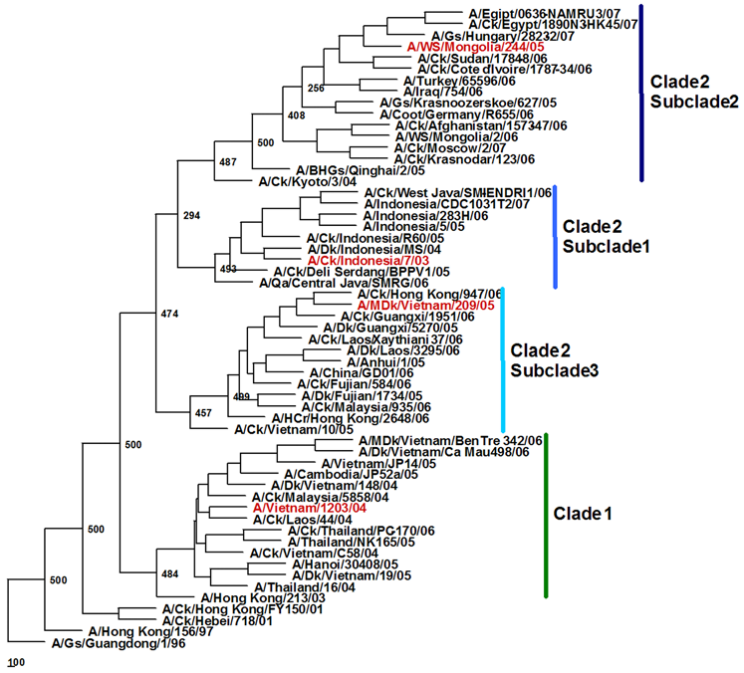
Phylogenetic relationships of the hemagglutinin (HA) gene of the influenza (H5N1) viruses. Sequences (nucleotides 77 to 1723) were analyzed by using the neighbor-joining method with 500 bootstraps. Phylogenetic tree was rooted to the HA gene of A/Goose/Guangdong/1/96 (H5N1) virus. H5N1 viruses used in this study are shown in red. Abbreviations: BHGs, bar-headed goose; Ck, chicken; Dk, duck; Gs, goose; HCr, house crow; MDk, muscovy duck; Qa, quail; WS, whooper swan.

Since 2003, H5N1 viruses has evolved rapidly and formed 2 major clades and multiple subclades based on the HA sequences phylogeny and antigenicity [18],[44]. In the present study we infected pigs with four H5N1 viruses representing clades 1 and 2, and subclades 2.1, 2.2 and 2.3 (Figure 1). Virological, histological and serological features of H5N1 infection in pigs were characterized and compared with those caused by swine H3N2 and H1N1 viruses.

## Results

### Characterization of H5N1 viruses used for pig infection

In order to characterize the variety of H5N1 viruses, 4 strains isolated from human, poultry and wild birds, A/Vietnam/1203/04 (VN/04), A/Chicken/Indonesia/7/03 (Ck/Indo/03), A/Whooper swan/Mongolia/244/05 (WS/Mong/05), and A/Muscovy duck/Vietnam/209/05 (MDk/VN/05) were chosen for this study. Phylogenetic analysis of the HA gene sequences of the H5N1 viruses showed that they represented clades 1 and 2, and subclades 2.1, 2.2 and 2.3 (Figure 1), respectively. Analysis of amino acid sequences of the HA revealed that all four viruses had conserved amino acid residues that retained the receptor binding of 2,3-NeuAcGal linkages predicted to confer affinity for avian cell surface receptors [17],[45]. The growth and infectivity of 3 viruses were comparable in MDCK cells and embryonating chicken eggs while titers of Ck/Indo/03 virus were lower (Table 1). All four H5N1 viruses killed chickens after intranasal inoculation and intravenous pathogenicity tests [46] indicating these viruses were highly pathogenic for chickens.

**Table 1:** Growth and pathogenicity of H5N1 viruses

Viruses	Virus growth a		Virus pathogenicity in		
	log_10_ EID_50_/ml	log_10_ TCID_50_/ml	Mice b	Ferrets c	Chickens d
Ck/Indo/03	8.5±0	6.9±0.6	Low	Low	High
VN/04	9.5±0	9.2±0.1	High	High	High
WS/Mong/05	9.0±0	8.8±0.3	High	Moderate	High
MDk/VN/05	9.3±0.35	9.4±0.3	High	Low	High

a All data are the mean±SD from three independent experiments.

b 90–100% mortality in groups of 10 mice after intranasal inoculation with virus dose of 10^3^ EID_50_ was considered as high pathogenicity; no mortality and disease signs after infection with similar virus dose was considered as low pathogenicity.

c Severe systemic disease with mortality developed after intranasal infection with virus dose of 10^6^ EID_50_ was considered as high pathogenicity; severe respiratory disease without mortality was considerate as moderate pathogenicity, and mild or asymptomatic respiratory infection without mortality as low pathogenicity.

d Intravenous pathogenicity tests [46] with all viruses killing 100% of inoculated chickens indicative of HPAI viruses.

Pathogenicity of H5N1 viruses was also characterized in mouse and ferret models. Intranasal inoculation of 8-weeks-old female BALB/c mice with 10^3^ 50% egg infective dose (EID_50_) of VN/04, WS/Mong/05, and MDk/VN/05 viruses resulted in systemic infection with 90–100% mortality. Ck/Indo/03 virus inoculated at the same dose produced mild lung infection without serious disease and mortality in mice. Only one H5N1 virus, VN/04, was highly pathogenic in 4–6-month-old female ferrets producing severe systemic disease with 100% fatality after intranasal inoculation of 10^6^ EID_50_ of virus. Infection of ferrets with 10^6^ EID_50_ of WS/Mong/05 virus resulted in severe respiratory disease without systemic infection and mortality, and was considered to be of moderate pathogenicity. Viruses, Ck/Indo/03 and MDk/VN/05 were considered as low pathogenicity in ferrets causing mild or asymptomatic respiratory infection in animals intranasally inoculated with 10^6^ EID_50_ of virus. The data on pathogenicity of H5N1 viruses are summarized in Table 1.

### Clinical signs after inoculation of pigs with H5N1 influenza viruses

**Figure 2:**
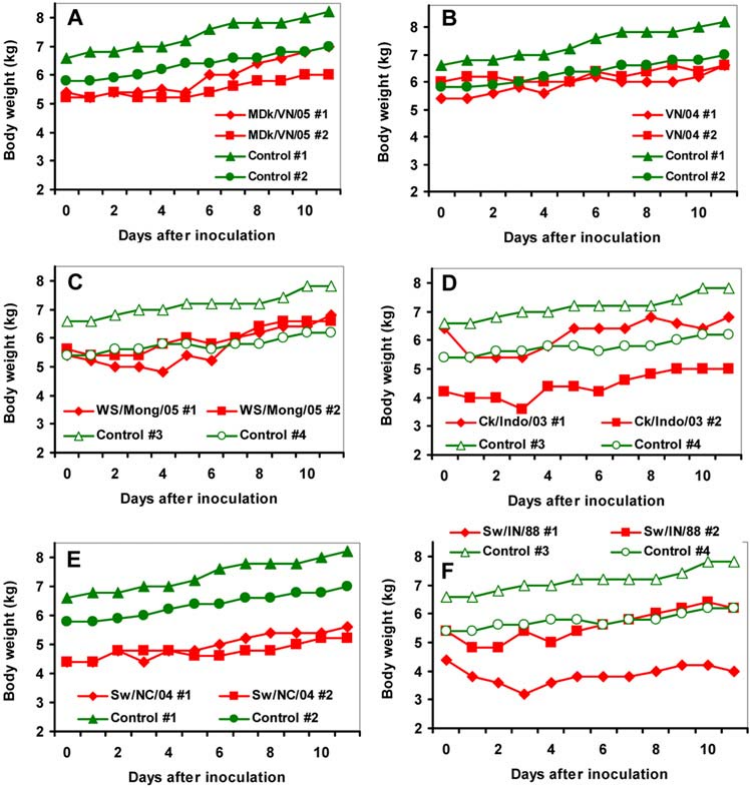
Pig weight changes during influenza virus infection. Shown are results for the two infected pigs for each virus treatment group (pigs #1 and #2); compared to one of two independent control groups (control pigs #1 and #2, or control pigs #3 and #4). H5N1 influenza viruses: MDk/VN/05 (A), VN/04 (B), WS/Mong/05 (C), and Ck/Indo/03 (D). Swine H3N2 (E) and H1N1 (F) influenza viruses.

Groups of 2–3-weeks-old piglets were inoculated intranasally with 10^6^ EID_50_ of H5N1 viruses. Controls that demonstrate the susceptibility of the animals to influenza virus infection, consisted of two groups of piglets that were intranasally infected with 10^6^ EID_50_ of swine H3N2, A/Swine/North Carolina/307408/04 (Sw/NC/04), and H1N1, A/Swine/Indiana/1726/88 (Sw/IN/88) influenza viruses. Body weight of infected pigs was measured daily and compared with that of mock-infected animals. No changes in food consumption or behavior were observed in inoculated animals. However, infection with swine influenza viruses produced slight lethargy and listlessness on day 1 after inoculation in one animal infected with H3N2 and in two animals infected with H1N1 viruses. Pigs inoculated with H5N1 viruses, MDk/VN/05 and VN/04, as well as those inoculated with H3N2 virus, Sw/NC/04, did not demonstrate remarkable differences in body weight compared to control animals (Figure 2A, B, E). However, weight loss of 5–15% was seen in pigs inoculated with WS/Mong/05 and Ck/Indo/03 H5N1 viruses on days 1–4 (Figure 2C, D). The most severe, up to 25%, decrease in weight was observed on day 3 in one animal infected with swine H1N1 influenza virus (Figure 2F).

### Shedding of H5N1 viruses

**Figure 3:**
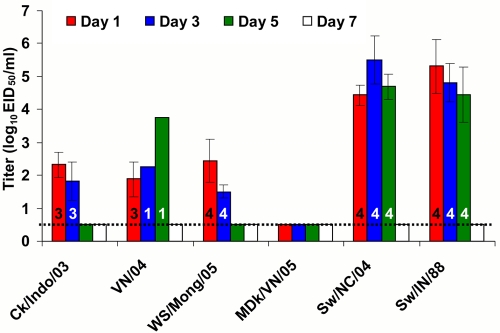
Virus titer in nasal swabs of pigs during influenza virus infection. Each data point represents the mean±SD virus titer (log_10_ EID_50_/ml of sample media) from pigs positive for influenza virus. Numbers of pigs sheding virus (of 4) are shown in each bar. The lower virus detection limit is 10^0.5^ EID_50_/ml.

To detect viruses and determine infective titers, nasal and rectal swabs were collected from infected animals. None of the influenza A viruses were detected in rectal swabs. Differences were observed in nasal excretion among the H5N1 viruses: WS/Mong/05 virus was detected in all 4 pigs on days 1 and 3 after inoculation, 3 of 4 pigs shed Ck/Indo/03 virus on days 1 and 3, VN/04 virus was detected in nasal swabs of 3 pigs on day 1 and only in 1 pig on days 3 and 5, while MDk/VN/05 virus was not detected in nasal swabs of inoculated pigs (Figure 3). Swine H3N2 and H1N1 viruses were detected in all inoculated pigs on days 1, 3, and 5 (Figure 3). In general, titers of H5N1 viruses in nasal samples collected on day 1 and 3 were similar, and were 2–3 log_10_ lower than those of swine H3N2 and H1N1 viruses which were detected at the similarly high titers (Figure 3).

### Organ tropism of H5N1 viruses

**Figure 4:**
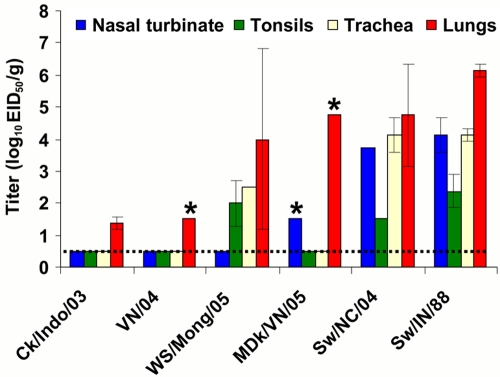
Virus titer in pig tissues on day 5 after virus inoculation. Each data point represents the mean and range virus titer (log_10_ EID_50_/ gram of tissue) from two pigs. The lower virus detection limit is 10^0.5^ EID_50_/g. *-virus was detected in organs of one pig.

To determine sites of viral replication, samples from 18 organs and tissues (see Materials and Methods) were collected from infected pigs on day 5 after virus inoculation. H5N1 influenza viruses as well as swine H3N2 and H1N1 viruses were detected only in tissues from the respiratory organs (Figure 4). All studied H5N1 viruses were detected in the lungs of inoculated pigs. Lung titers of WS/Mong/05 and MDk/VN/05 (detected in one of two pigs) viruses were high and comparable with those of swine H3N2 and H1N1 viruses, while lung titers of Ck/Indo/03 and VN/04 (detected in one of two pigs) viruses were remarkably lower. MDk/VN/05 virus was also detected in nasal turbinate of one infected pig. The replication sites and titers of WS/Mong/05 virus, which was detected in lungs, trachea and tonsils, were close to those of swine H3N2 and H1N1 influenza viruses which were detected at high titers in upper and lower respiratory tract (Figure 4).

### Histopathological findings

**Figure 5:**
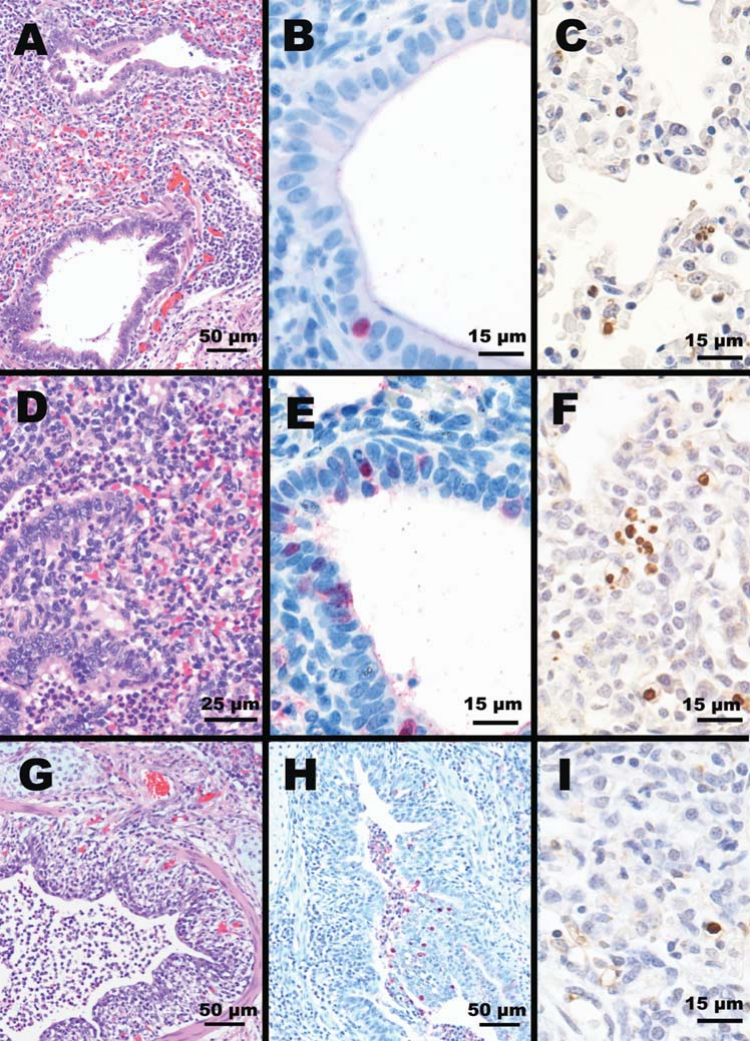
Histopathologic findings in lungs of pigs on day 5 after virus inoculation. (A) Moderate lymphocytic bronchiolitis with slight intra-luminal cellular debris (HE staining) and (B) viral antigen reaction in a single bronchiolar cell (IHC staining) in the lungs of pigs infected with VN/04 (H5N1) virus. (D) Moderate bronchioalveolitis with moderately cellular debris in the airway of bronchioles and alveoli (HE staining) and (E) marked viral antigen reaction in bronchiolar cells (IHC staining) in the lungs of pigs infected with WS/Mong/05 (H5N1) virus. (G) Severe bronchitis characterized by degeneration and necrosis of bronchi epitheliums with severe intra-luminal cellular debris (HE staining) and (H) prominent viral antigen reaction in the bronchial epitheliums and necrotic cellular debris (IHC staining) in the lungs of pigs infected with Sw/NC/88 (H3N2) virus. Apoptosis frequently observed in proliferating leukocytes and macrophages in the lungs of pigs infected with (C) VN/04 and (F) WS/Mong/05 H5N1 viruses (TUNEL assay). (I) TUNEL assay of lungs from pig infected with Sw/NC/88 (H3N2) virus–a single cell affected with apoptosis.

Gross and microscopic lesions were observed in the respiratory tract of all pigs inoculated with either avian or swine influenza viruses. The extent and character of the lesions were variable between pigs in a group, and among virus treatment groups. When present, lesions were most often observed in the lungs. H5N1-inoculated pigs had minimal to mild gross lesions. Microscopic lung lesions included mild to moderate bronchiolitis and alveolitis found on day 5 post inoculation. In addition, moderate lymphocytic infiltration around peribronchiolar and perivascular areas (Figure 5A), mild degeneration to necrosis of bronchiolar epithelium, and moderate necrotic cell debris in the airways of bronchioles and alveoli (Figure 5D) were observed. The upper airways and bronchi were spared lesions. Immunohistochemically, viral antigen was detected in bronchiolar epithelium (Figure 5B and E). On day14 post-inoculation, there was no histological lesion in any visceral organs including lungs. Viral antigens were detected only in the lung of pigs inoculated with VN/04, WS/Mong/05 and MDk/VN/05 viruses which were also positive on virus isolation. Based on gross and microscopic lesions, the pathogenicity of the H5N1 viruses could be ranked in the following order: WS/Mong/05, VN/04, MDk/VN/05, and Ck/Indo/03.

By comparison, the respiratory lesions from pigs infected with swine viruses (H3N2 and H1N1) were more severe and more extensive than those from pigs infected with H5N1 viruses. The lungs from pigs infected with swine viruses on day 5 had severe bronchointerstitial pneumonia characterized by severe degeneration and necrosis of bronchial epithelium and accumulation of necrotic cellular debris within airway lumens (Figure 5G). Consistently, viral antigen was conspicuously detected to bronchial epithelial linings and cellular debris in the airway (Figure 5H). In addition, the nasal cavities of pigs infected with H3N2 swine virus showed mild vacuolar degeneration and necrosis of mucosal epithelium; also, severe tracheobronchitis was observed in both H3N2- and H1N1-infected pigs. Mild lymphocytic infiltration around peribronchial areas was still evident in the lungs of swine viruses-infected pigs on day 14 post-inoculation. However, no viral antigen was detected in any tissues or organs on day 14 by immunohistochemistry.

Recently, human infection with H5N1 viruses was reported to produce apoptosis in alveolar epithelial cells and leucocytes in the lungs [47]. To determine whether H5N1 viruses result in similar lesions in pigs, lung sections adjacent to those confirmed for presence of viral antigen from animals infected with H5N1 and H3N2 influenza viruses were stained by TUNEL assay. Apoptosis was frequently observed in proliferating cells, most likely leukocytes and macrophages in the lungs of pigs infected with all four H5N1 viruses. In general, the amount of cells with apoptosis correlated with the severity of lesions produced by H5N1 viruses in the lungs. The greatest numbers of stained cells were observed in the lung samples from pigs infected with VN/04 (Figure 5C) and WS/Mong/05 (Figure 5F) viruses. In contrast, very small, almost negligible numbers of cells with apoptosis, comparable with those in uninfected control lung samples, were observed in animals infected with swine H3N2 virus (Figure 5I).

### Antibody response after inoculation with H5N1 viruses

To confirm the infection, blood samples collected from pigs prior to and two weeks after virus inoculation were examined in hemagglutination inhibition (HI) and virus neutralization (VN) tests with the homologous viruses to assess the seroconversion. Pre-infection sera lacked antibodies detectable by HI or VN test with H5N1 viruses, but small, almost negligible antibody titers (presumably of maternal transfer origin) were observed only in the HI test when using swine H1N1 and H3N2 viruses as the HI test antigen (Table 2). By contrast, all pigs challenged with H5N1 viruses Ck/Indo/03 and WS/Mong/05 had specific antibodies in HI and VN tests (Table 2) on day 14 post-inoculation. High antibody titers were also observed in both HI and VN tests in serum from 1 pig (of 2) inoculated with H5N1 VN/04 virus, and very low titers of virus-neutralizing antibodies were detected in 1 pig (of 2) inoculated with H5N1 MDk/VN/05 virus. All animals seroconverted after intranasal inoculation with swine H1N1 and H3N2 viruses as evident by high levels of antibodies in both HI and VN tests using the challenge viruses (Table 2).

**Table 2:** Pig pre- and post-exposure serum antibody titers

Pigs infected with:	Antibody titers to challenge virus			
	Pre-infection sera		Post-infection sera a	
	HI test	VN test	HI test	VN test
Ck/Indo/03	<10	<20	10–160 (2)	80–1280 (2)
VN/04	<10	<20	320 (1)	1280–5120 (1)
WS/Mong/05	<10	<20	10–80 (2)	80–320 (2)
MDk/VN/05	<10	<20	<10	20–40 (1)
Sw/NC/04	10–20	<20	160–320 (2)	640–2560 (2)
Sw/IN/88	20	<20	640 (2)	640–1280 (2)

a The number of pigs (of 2) positive for antibodies against the challenge virus is shown in brackets.

### Exposure of pigs to H5N1 virus through consumption of meat from infected chickens

The consumption of raw or undercooked infected bird meat or other products is one of potential means of transmission of H5N1 HPAI virus to humans [11],[24] and several animals belonging to order *Carnivora* [33]–[37],[48]). To model this potential route of infection, piglets in one group of 4 were fed breast and thigh meat from chickens that died from infection with WS/Mong/05 H5N1 virus. The meat was chopped into small pieces approximately 4 cm×2 cm×0.5 cm in size and mixed with a limited amount of pelleted diet. Each animal consumed approximately 100 g of meat with infective virus titer 10^8^ EID_50_/g. No disease signs such as significant weight loss, changes in food consumption or behavior abnormalities were observed in exposed pigs during the 14 day observation period. Virus was detected in nasal swabs from 2 of 4 pigs on day 3 only (Table 3). No virus was detected in rectal swabs. Two pigs were euthanatized on day 5 after meat consumption and samples from 18 organs and tissues (see Materials and Methods) were harvested to determine virus distribution and histological lesion. Infective virus was detected in nasal turbinate and tonsils of both animals (Table 3). Microscopically, the organs or tissues lacked histological lesions and viral antigen was not demonstrated. However, virus-neutralizing antibodies to WS/Mong/05 virus were detected in serum samples from both pigs collected on day 14 after consumption of infected meat indicating infection had occurred (Table 3).

**Table 3:** Exposure of pigs to WS/Mong/05 H5N1 virus through consumption of meat from infected chickens

Virus titers in nasal swabs on day 3 (log_10_ EID_50_/ml) a	Organ titers on day 5 (log_10_ EID_50_/ml) b		Serum antibody titer (VN test) to WS/Mong/05 virus	
	Nasal turbinate	Tonsils	Pre-exposure	Post-exposure
2.63±0.49	2.63±0.18	2.88±0.18	<20	80

a Virus was detected in nasal swabs from 2 of 4 pigs on day 3 only.

b Virus was detected only in nasal turbinate and tonsils of 2 pigs killed on day 5 after consumption of infected chicken meat.

## Discussion

It was proposed that expression of sialic acid receptors for human and avian influenza viruses on epithelial cells of the trachea [5], renders pigs susceptible to infection with both types of influenza viruses [3],[4],[49],[50]. Influenza viruses from pigs can be transmitted to humans [3],[4],[51],[52] as well as human viruses and human/pig gene reassortant viruses can be isolated from pigs [53]. Recently, a H2N3 swine influenza subtype was reported in the USA. It was an avian/swine reassortant virus that was pathogenic in pigs and mice, and was transmitted among swine and ferrets [54]. Thus, it seems possible to propose that H5N1 highly pathogenic avian influenza viruses, which spread through Eurasia and Africa, could reassort in pigs with currently circulating human influenza viruses and/or adapt to efficient transmission in humans, and acquire a pandemic potential.

In this study we characterized in a pig model virological, histological, and serological features of infection with H5N1 HPAI viruses representing major HA phylogenetic and antigenic clades and subclades of currently circulating H5N1 viruses, i.e. clade 1 and clade 2, subclades 2.1, 2.2 and 2.3 (Figure 1). These viruses differed in their pathogenicity in well characterized mammalian models, i.e. mice and ferrets (Table 1). Three of the H5N1 viruses replicated systemically in mice and caused high mortality, but only one caused high mortality in ferrets. In contrast all four viruses had similar low pathogenicity in intranasally inoculated pigs. In pigs, the H5N1 viruses replicated only in the respiratory tract with no evidence of systemic infection. All four H5N1 viruses replicated in lungs of inoculated pigs and resulted in moderate or mild bronchiolitis and alveolitis. WS/Mong/05 and MDk/VN/05 H5N1 viruses were also detected in upper respiratory tract tissues (trachea) and tonsils. In contrast to the other studied H5N1 viruses, titers and organ distribution of WS/Mong/05 (clade 2, subclade 2 of H5 HA) in inoculated pigs were most similar to those seen with the swine H3N2 and H1N1 viruses.

With the exception of severity, the type and location of virus-induced lesions in the lower respiratory tract of H5N1-infected pigs were similar to those observed in humans [11]. However, viral antigens in pigs infected with H5N1 viruses were detected immunohistochemically in bronchiolar epithelial cells only, in contrast to reported patterns of H5N1 virus attachment to type II pneumocytes in pig, ferret and human lungs [55],[56], and human cases there viral antigens were observed in ciliated and nonciliated tracheal epithelial cells [57] and type II pneumocytes [57],[58].

Interestingly, lung infection of pigs with H5N1 viruses resulted in apoptosis in proliferating leucocytes and macrophages while infection with swine influenza viruses did not, although greater severity of histological lesions were noted with swine influenza virus infections. As we did not find apoptosis in alveolar epithelial of H5N1-infected pigs, our finding only partially resembles the observations of Uiprasertkul and co-authors [47] where frequent apoptosis was identified in alveolar epithelial as well as in proliferating leucocytes in lungs of humans who died in the course of H5N1 virus infection. Our observation suggests tissue pathogenesis of avian H5N1 and swine H3N2 viruses in pigs might be different and such differences could underlay the lower efficacy of replication of H5N1 HPAI viruses in pigs.

Serological studies with pigs showed very low pre-challenge levels of antibodies detectable only in HI test with swine H1N1 and H3N2 influenza A viruses (Table 2). Such antibodies most likely represented maternal transfer. Studies in a mouse model demonstrated that antibodies to human influenza A viral neuraminidase N1 could partially protect animals from lethal infection with H5N1 viruses [59]. This observation raised a concern that maternal antibodies to N1 could influence the course of H5N1 infection in pigs and was the reason for including H1N1 swine influenza virus challenge. However, the antibodies to H1N1 virus did not interfere with H1N1 swine influenza virus replication in pigs challenged with Sw/IN/88. Furthermore, the antibodies to H3N2 virus did not inhibit replication of H3N2 swine influenza virus. From the current experiments, the detection of H5N1 virus replication and presence of specific serum antibodies against H5N1 virus implies that the low levels of H1N1 antibodies did not significantly interfere with H5N1 virus replication in the respiratory tract of pigs.

Overall, the results of this study indicate that commercial piglets can support replication of H5N1 HPAI viruses, but their susceptibility to infection is low. The course of H5N1 virus infection in pigs was almost asymptomatic which could delay or prevent diagnosis of H5N1 infection in pigs. The infected pigs shed H5N1 virus, but the viral titers were lower and time of shedding was shorter in comparison with H1N1 and H3N2 swine influenza viruses. In addition, there was individual strain variation following infection of pigs with different H5N1 viruses. Intranasal inoculation with MDk/VN/05 (subclade 2.3) produced infection detected by a single seroconversion and no virus recovery from nasal cavity, while inoculation with VN/04 virus (Clade 1) produced a seroconversion in one of two pigs and low titers of virus were found in nasal cavity on day 1 in 3 pigs. By contrast, the virus isolated from wild migratory birds, WS/Mong/05 (subclade 2.2) infected all pigs in the group, and tissue tropism and titers of this virus were similar to those of swine influenza viruses. However the individual susceptibility of pigs to influenza infection is highly variable. As the number of animals in this study was minimal and not suitable for statistical evaluation, we can not exclude that differences observed among the H5N1 viruses are the result of variations in individual susceptibility of pigs.

In addition, consumption of chicken meat infected with high titers of virus (10^10^ EID_50_/pig) produced a subclinical infection in pigs. The presence of virus in tonsils and the upper respiratory tract suggests that contact between the infected meat and oropharynx initiated infection, most likely through the tonsil. During the 2003 H7N7 poultry outbreak in the Netherlands, infections were detected in pigs on farms with infected poultry, and in some instances, the pigs had been fed broken eggs from the infected chickens [60]. This suggests consumption of infectious virus in raw or uncooked contaminated product can potentially transmit the virus to mammals.

The main question resulting from the current study is why this experimental mammalian host has lower susceptibility to infection as compared to ferrets and mice? It is possible, that further detailed studies of immunopathogenesis of H5N1 infection in pigs will reveal the mechanism of such resistance. This knowledge could be extremely useful for new approaches for treatment of H5N1-induced disease and for the design of new antivirals.

## Materials and Methods

### Viruses and cells

H5N1 viruses A/Chicken/Indonesia/7/03 (Ck/Indo/03) and A/Whooper swan/Mongolia/244/05 (WS/Mong/05) were isolated at Southeast Poultry Research Laboratory from field samples by passage in 10-day-old embryonating chicken eggs. Human isolate of H5N1 highly pathogenic avian influenza virus, A/Vietnam/1203/04 (VN/04) was obtained from World Health Organization collaborating laboratories in Asia through National Institute of Allergy and Infectious Diseases, National Institutes of Health (NIAID, NIH), Bethesda, MD, USA. H5N1 virus A/Muscovy duck/Vietnam/209/05 (MDk/VN/05) was provided by Dr. Nguyen Van Cam from National Center for Veterinary Diagnosis, Hanoi, Vietnam. Swine H3N2 virus A/Swine/North Carolina/307408/04 (Sw/NC/04) and H1N1 virus A/Swine/Indiana/1726/88 (Sw/IN/88) were obtained respectively from National Veterinary Services Laboratories, Ames, Iowa, USA and the University of Wisconsin, Madison, Wisconsin, USA. Virus stocks were produced by passage in 10-day-old embryonating chicken eggs. H5N1 viruses Ck/Indo/03, WS/Mong/05, MDk/VN/05 were the 2^nd^ chicken embryo passage and VN/04 isolate was the 4^th^ chicken embryo passage after isolation. The allantoic fluid from infected eggs was harvested, divided into aliquots, and stored at –70°C until it was used for experiments. The infectivity of stock viruses was determined in 10-day-old embryonating chicken eggs and in Madin-Darby canine kidney (MDCK) cells according to standard procedures. The 50% egg infective dose (EID_50_) and the 50% tissue culture infectious dose (TCID_50_) values were calculated by the Reed-Muench method [61]. All experiments with live H5N1 viruses were performed in a biosafety level 3 agriculture (BSL-3AG) biocontainment facility, and all personnel were required to use respiratory protection when working with live viruses or infected animals.

MDCK cells were obtained from the American Type Culture Collection (Manassas, VA) and were cultured in Dulbecco's Modified Eagle's Medium supplemented with 5% fetal bovine serum.

### Experimental infection of pigs

Two to three weeks-old male castrated piglets (Landrace×Large White cross) were purchased from a local commercial farm. The pigs did not receive any vaccines on the production farm. In the BSL-3AG animal laboratory facilities pigs were housed in HEPA-filtered isolation units at a constant 27°C. Three to five days were taken to acclimatize animals to the facility. Piglets were feed with commercially available pelleted diet in amounts prescribed by the manufacturer to fulfill all dietary needs. Animal experiments were conducted according to the protocols approved by the Institutional Animal Care and Use Committee based on the applicable laws and guidelines. Each virus treatment group consisted of 4 pigs that were anesthetized with the intramuscular injection of ketamine (20 mg/kg) and xylazine (2 mg/kg) mixture and inoculated intranasally with virus dose of 10^6^ EID_50_ in 2 ml of PBS (1 ml in each nostril). Control pigs (two separated groups of 2 animals) were inoculated with 2 ml of sterile PBS. The pigs' body weights, temperatures and feed consumption were monitored daily, starting 1 day before inoculation and ending on day 11 after inoculation.

### Collection of samples, virus detection and titration

Nasal and rectal swabs were collected 3 or 4 days before the infection and on day 1, 3, 5, 7, 9, and 11 after virus inoculation. Swabs were tested in 10 day-old embryonating chicken eggs to detect and titer virus (lower detection limit, 10^0.5^ EID_50_/ml). Before the titration, each sample of allantoic fluid that was positive in a hemagglutination test was confirmed to be influenza A virus positive by solid phase ELISA assay (BinaxNow, Scarborough, ME). Virus titers were expressed as log_10_ EID_50_ per 1 ml of swab media. Two pigs from each group were euthanatized on day 5 after virus inoculation and the following organs and tissues were collected during the necropsy: nasal turbinate, tonsils, trachea, lungs, olfactory bulbs, brain (transverse section through mid-cerebrum, thalamus, cerebellum/pons and medulla oblongata), heart, whole blood (collected in sterile PBS to prevent clotting), spleen, liver, stomach, pancreas, small intestine (upper part of duodenum and middle part of jejunum), large intestine (rectum), kidney, adrenal glands, diaphragm, and skeletal muscle. Tissues were weighed and grounded in sterile PBS with antibiotics to prepare 10% homogenates. Samples were injected into 10 day-old embryonating chicken eggs for virus detection and titration as described above.

### Serological assays

Pigs were bled one day before and on day 14 after virus inoculation. To destroy non-specific inhibitors, serum samples were heat inactivated at 56°C for 30 min and treated with 10% chicken red blood cell (CRBC) for 60 min at 4°C. Serum antibody titers were determined in hemagglutination inhibition (HI) test with 0.5% CRBC and virus neutralization test (VN) in MDCK cells according to standard procedures described previously [29]. Virus infective dose of 100 TCID_50_ was used for VN test; MDCK cells were incubated for 72 h at 37°C.

### Histological analysis and immunohistochemistry

Tissues samples collected at necropsy on day 5 and 14 after virus inoculation were preserved in 10% neutral buffered formalin. After fixation, the tissues were routinely processed and embedded in paraffin. Sections were cut at 5 µm and stained with hematoxylin and eosin. Duplicate sections were cut and immunohistochemically stained using a mouse-derived monoclonal antibody (P13C11) specific for type A influenza virus nucleoprotein antigen as the primary antibody. The procedures used to perform the immunohistochemistry followed those previously described [62],[63]. Fast red was used as the substrate chromagen, and slides were counterstained with hematoxylin. Two to five sections of each organ was stained with hematoxylin and eosin and their immunohistochemically stained duplicates were analyzed.

### Apoptosis analyses

Lung sections from infected and control animals were analyzed for apoptosis by using the terminal deoxynucleotidyl transferase-mediated dUTP-biotin nick end-labeling (TUNEL) assay (*In Situ* Cell Death Detection Kit, POD, Roche, Mannheim, Germany), according to the protocol provided by the manufacturer, and slides were counterstained with hematoxylin.

### Virus sequencing and phylogenetic analysis

Viral RNAs were extracted from the allantoic fluid by the use of Trizol LS reagent (Invitrogen Inc., Carlsbad, CA). Standard reverse transcription-PCR was performed by use of a One-Step RT-PCR kit (QIAGEN, Valencia, CA) with primers specific for influenza virus HA of H5 subtype. The primer sequences and amplification conditions used are available upon request. The PCR products were separated in an agarose gel by electrophoresis, and amplicons of the appropriate sizes were subsequently excised from the gel and extracted by use of a QIAGEN gel extraction kit. Sequencing was performed with a PRISM Ready Reaction DyeDeoxy Terminator cycle sequencing kit (Perkin-Elmer, Foster City, CA) run on a 3730 automated sequencer (Perkin-Elmer). DNA sequences were completed by using the Lasergene sequence analysis software package (DNAStar, Madison, WI). The nucleotide sequences of WS/Mong/05 and MDk/VN/05 HA genes have been deposited in the GenBank database under accession numbers EU723707 and EU723708 respectively.

Reference sequences of the HAs of H5 subtype were uploaded from the Influenza Sequence Database at Los Alamos National Laboratory (www.flu.lanl.gov) [64]. Sequences (nucleotides 77 to 1723) were compared by ClustalW alignment algorithm by using BioEdit Sequence Alignment Editor (www.mbio.ncsu.edu/BioEdit/bioedit.html). To estimate phylogenetic relationships, we analyzed nucleotide sequences by the neighbor-joining method with 500 bootstraps by using PHYLIP (the PHYLogeny Inference Package) version 3.65 (http://evolution.gs.washington.edu/phylip.html).
